# Epidemiology of Urinary Schistosomiasis among School Children in the Alsaial Alsagair Village, River Nile State, Sudan

**Published:** 2017

**Authors:** Yassir SULIEMAN, Randa E. ELTAYEB, Theerakamol PENGSAKUL, Azzam AFIFI, Mohamed A. ZAKARIA

**Affiliations:** 1. Dept. of Zoology, Faculty of Science and Technology, University of Shendi, Shendi, Sudan; 2. Faculty of Medical Technology, Prince of Songkla University, Hat Yai, Songkhla, Thailand; 3. Dept. of Zoology, Faculty of Science and Technology, Omdurman Islamic University, Omdurman, Sudan; 4. Dept. of Biology, Faculty of Education, University of Nyala, Nyala, Sudan

**Keywords:** Prevalence, Intensity, Urinary schistosomiasis, Sudan

## Abstract

**Background:**

Schistosomiasis is a debilitating disease, infects millions of people in tropical and subtropical regions. This study assessed the situation of the urinary schistosomiasis among schoolchildren of the Alsaial Alsagair village, Sudan.

**Methods:**

A cross-sectional survey was carried out in 2016, in the Alsaial Alsagair village, and 385 samples of urine were collected and processed using the centrifugation/sedimentation technique.

**Results:**

The overall prevalence and intensity of the disease among the pupils studied were 1.82% and 40.1 eggs/10ml urine, respectively. The prevalence of infection among male pupils was 3.1%, and for females was 0.52%, while the intensity among males was 42.3 eggs/10ml and for females was 27 eggs/10ml urine. No significant difference in the disease prevalence between the 7–10 yr age group and the 11–14 yr age group was found, while the over 14 yr age group was found to be free from infection. However, a considerably higher intensity of the parasite was found among the 7–10 yr age group compared to the other age group infected. A high prevalence and intensity of infection was observed among pupils who were active in swimming and working in the fields. Most of the pupils interviewed were found to be unaware of the disease and the prevalence and intensity of the infection was found to be higher among the group who were aware of the disease.

**Conclusion:**

Findings show the need for an integrated control program against urinary schistosomiasis including the treatment of all infected children and the implementation of a health education program.

## Introduction

Schistosomiasis, also known as Bilharzia, is a chronic and debilitating disease caused by trematode flukes of the genus *Schistosoma* (1–3). There are four main species of schistosomes responsible for two forms of human schistosomiasis; intestinal, caused by *S. mansoni*, *S. japonicum* and *S. intercalatum* and urogenital, caused by *S. haematobium* (3–6). These parasites are estimated to infect more than 230 million people in rural agriculture and peri-urban areas of tropical and subtropical regions (7, 8), and about 500 million other people at risk of infection (5). Moreover, according to the WHO’s disability-adjusted-life-years (DALYs) estimation, schistosomiasis results in losses of up to 4.5 million annually (9). The transmission of schistosomiasis has been reported in 78 countries (10). It occurs only in places where fresh water snail vectors are present and where there is contact between the population and infested water (11, 12).

Various socio-epidemiological factors are responsible for the transmission and level of infection of the disease (13, 14). Among these factors are the following: distance from transmission site, migration and the emergence of new foci, urbanization, socioeconomic status, sanitation, water supply patterns and level of fecal or urine contamination of water sources (15). Those at high risk of infection are people involved in fishing activities, farming, bathing, canoeing, swimming and those who may handle infected snail hosts while collecting edible foodstuffs (9, 16).

In Sudan, and because of the large expansion of water resource projects, waterborne diseases, such as schistosomiasis has been increased. In this country, the first case of schistosomiasis was reported (17), 17% of the children in Khartoum Primary School suffering from urinary schistosomiasis. Since 1919, the disease has been discovered in the northern part of the country (18) and later it was reported in other parts including Eastern and Western Sudan (19–21), the Gezira Agricultural Scheme (22, 23), the Rahad Agricultural Scheme (24) and the Gunaid Sugar Cane Scheme (16, 25). Moreover, new foci of disease transmission in the country have been identified.

“Children should be the first target group for intervention because of the detrimental effects of the disease on their growth and development” (26). Therefore, the objective of the present study was to investigate the situation of urinary schistosomiasis infection among school-children in the Alsaial Alsagair village at Al Matama Locality, River Nile State, Sudan. The study’s findings will provide a baseline of information used in future control strategies.

## Materials and Methods

### Study area

This study was carried out as a cross-sectional parasitological survey, from Jan to Mar 2016 in the Alsaial Alsagair village (16 °42′11″N and 33 °24′8″ E), Almatama Locality, River Nile State, Sudan. This village is very near to the eastern bank of the River Nile (∼2 km) and has an estimated population of 1485 inhabitants, all from the same ethnic group, the majority of whom earn their livelihood from subsistence farming. The village enjoys a good piped water supply from wells, and there are a health center and two elementary schools; one for boys, the other for girls, from which the study samples were collected. In addition, there is an electrical supply for most of the houses which are built of mud-brick or red-brick. The area has an average annual temperature ranging from 20–41 °C and a mean precipitation of 29.3mm.

### Ethical considerations

Permission to conduct this study was obtained from both, the Almatama Locality Health Office and the Education Office. The objectives of the study were explained to the two elementary schools’ administrators, staff members and to the pupils to encourage their participation in the study. All the pupils found on microscopic examination to be infected with schistosomiasis were treated under medical supervision.

### Sample collection and examination

Pupils from the two selected elementary schools (194 boys and 191 girls) were requested to provide samples of urine as well as some demographic data and information such as their age, knowledge about schistosomiasis, previous infection with the disease and treatment and water contact habits, and they were also asked to provide details of their guardians’ occupations and monthly income. Each individual was given a clean-labeled 60ml plastic container in which to collect their urine and they were instructed to half fill the container with their urine sample. The containers were placed in zipped plastic bags, kept in an ice-cooled box and were transported to the Laboratory of Zoology, University of Shendi within two hours of collection. They were then microscopically examined using the simple centrifugation/sedimentation technique (16). The prevalence and intensity of infection were calculated in accordance with the procedure set out in Bush et al. (27).

### Data Analysis

Data analysis was performed using the statistical software program, SPSS 16.0 (Chicago, IL, USA) for Windows. A chi-square test was used to determine the level of significance in the differences found in the prevalence rates, while paired sample *t*-tests were used to detect any significant differences in the intensity of infection. One-way ANOVAs were used for other comparisons involving more than two variables. Values were considered significant when *P*<0.05.

## Results

### The overall prevalence and intensity of urinary schistosomiasis infection

Samples were successfully collected from all 385 pupils requested to provide urine samples (194 boys and 191 girls). The overall prevalence of *S. haematobium* infection among pupils at the two study sites was 1.82%, while the mean intensity among those infected was 40.1eggs/10ml urine. A higher prevalence and intensity of infection was observed among pupils at the boy’s school than among the pupils at the girl’s school (Table 1) and the differences were found to be significant at the *P*<0.05 level.

**Table 1:** Infection parameters of *S. haematobium* according to some associated factors, among Alsaial Alsagair schools children (n = 385), River Nile State, Sudan

**Variables**	**Number examined (Frequency %)**	**Prevalence %**	**Mean intensity ± SE (eggs/10 ml)**
Study site			
Alsaial school (boys)	194(50.4)	3.10	42.3±10.5
Alsaial school (girls)	191(49.6)	0.52	27±0.0
Water contact habits			
Swimming	15(3.9)	13.3	60±33
Planting crops	46(11.9)	4.3	40±0.0
Playing	45(11.7)	2.22	27±0.0
Collecting fodder	41(10.6)	0.0	0.0
Visiting	29(7.5)	0.0	0.0
Drinking	6(1.6)	0.0	0.0
Crossing	3(0.8)	0.0	0.0
No contact	200(51.9)	1.0	27±0.0
Guardians’ occupation			
Farming	160(41.6)	1.9	31.3±4.3
Trading	63(16.4)	1.6	93±0.0
Others	162(42.1)	1.9	31.3±4.3
Guardians’ income			
High	8(2.1)	12.5	27±0.0
Moderate	363(94.3)	1.4	42.8±12.8
Low	14(3.6)	7.1	40±0.0
Aware of the disease			
Yes	142(36.9)	3.5	45.4±12.24
No	243(63.1)	0.8	27±0.0
Previous infection/treatment			
Yes	37(9.6)	8.1	35.7±4.3
No	348(90.4)	1.1	43.5±16.5

### Infection parameters by water contact habits

The highest prevalence and intensity of infection with the disease was observed among pupils who reported swimming, followed by those who reported going to the fields to work or to play in the canals. However, pupils who had no water-contact activities were infected with urinary schistosomiasis (Table 1). The variations in the two infection parameters in relation to the categories of water contacts were statistically significant at the *P*<0.05 level.

### Infection parameters by guardians’ occupation and income level

While pupils whose guardians were farmers were found to have a high prevalence of infection, those whose guardians were traders were found to have the highest intensity of infection (Table 1). On the other hand, those pupils whose guardians had a high monthly income were also found to be subject to a high prevalence of infection, while those whose guardians had moderate and low monthly incomes had higher intensities of infection.

### Infection parameters by history of infection

Most of the pupils interviewed indicated that they had not previously been infected, while only a few said that they had experienced previous infection treated already (Table 1). Based on prevalence of infection, the pupils previously infected had a significantly higher prevalence than the group that had not previously been infected (*P*<0.05). However, the intensity of infection was found to be higher in the group not previously infected (Table 1).

### Infection parameters by awareness of the disease

Though some of the pupils interviewed were aware of the disease, most of them did not know about schistosomiasis or its transmission mode (Table 1). However, 71.4% of those infected, were aware of their infection status, and the prevalence and intensity of infection with the disease were found to be higher among the group who were aware of the disease.

### Infection parameters by age-groups

There was no significant difference in the prevalence of S. *haematobium* infection between the 7–10 yr age group and the 11–14 yr age group, while the >14 yr age group of was found to be free from infection. On the other hand, a significantly higher intensity of parasite infection was found among the 7–10 yr age group compared to the other infected age group (*P*<0.05) (Table 2).

**Table 2:** Infection parameters of *S. haematobium*, according to the study participants age-groups, Almatama Locality, River Nile State, Sudan

**Age group (yr)**	**Number examined (Frequency %)**	**Prevalence %**	**Intensity±SE (Eggs/10ml)**
7 – 10	207(53.8)	1.9	43.5±16.5
11 – 14	171(44.4)	1.8	35.7±4.3
> 14	07(01.8)	0.0	0.0
Overall	385(100)	1.82	40.1±9.11

## Discussion

In the present study, the overall prevalence and intensity of infection of urinary schistosomiasis was determined in the elementary school pupils of the Alsaial Alsagair village, Almatama Locality, Sudan, and found to be lower than that reported in other areas including in Cameroon (28), Southern Sudan (29), Mali (30), Nigeria (31) and Sudan (32).

This finding could be explained by the lower level of subjects’ exposure to infected water bodies, or to previous deworming conducted, or even to sample size. In addition, better hygiene, access to treatment, and less occupational exposure to infested water may reduce the chance of infection at a community level.

Regarding the gender of the study participants, the prevalence and intensity of *S. haematobium* infection were higher among the male pupils than the females. The relationship of prevalence and intensity of schistosomiasis infection to human gender has been previously studied in many endemic areas. The prevalence has been reported as being higher in males than in females in Egypt (33, 34), Senegal (35), Uganda (36), Brazil (37), Ghana (38, 39), Nigeria (31, 40), and Sudan (25, 32). However, in Gambia, the prevalence and intensity of infection were found to be similar in males and females despite the apparent differences in exposure to infection (41). Gender variations in schistosomiasis infection might be attributed to the activities engaged in by males and females in certain communities. Although fetching water from canal systems is a female job in many communities, females do not swim and rarely wash or bath in the canals near human dwellings, whereas males usually swim, wash and/or bathe in the canals and are therefore more likely to be exposed to the infective stage of the parasite and hence to infection than are females (42, 43). Moreover, agricultural activities in the fields are mainly carried out by males in many communities due to socio-cultural beliefs.

In the present study, the prevalence of *S. haematobium* infection tended to increase with the age of the participants, and children in the 7 to 14 yr age range were the most infected group. This finding is consistent with previous reports from different countries (2, 44–46). This could be explained by the fact that children of this age are more mobile and often go to the fields near the village either to assist their relatives in agricultural activities or to swim and play in the canals, which are likely to be contaminated with the infective stages of schistosomiasis or other parasitic diseases. Moreover, there are reports of a high prevalence of schistosomiasis infection among children who acquire the infection by being frequently exposed to fresh water containing the infective stage of the disease (47, 48). In addition, in most endemic areas, the prevalence and intensity of infection with schistosomiasis increase with age up to 10–20 yr, followed by a decline in older ages (49, 50).

In the present study, most of the pupils interviewed indicated that they had not previously been infected with urinary schistosomiasis, but unfortunately, when they were microscopically examined, they were found to have contracted the disease. This finding is most likely because the participants were either not aware of the disease or were lightly infected. Lack of information about the causes of parasitic diseases and ways of preventing their transmission/infection may contribute immensely to the status of the disease within communities. The highest prevalence and intensity of infection with the disease observed in this study was among those pupils who were active in swimming followed by those who went to work in the fields or played in the canals. This is because swimming or bathing necessarily requires extensive exposure to bodies of water resulting in high prevalence rates and high intensities. Contact with bodies of water and workings in irrigated agricultural fields are significantly associated with the risk of infection with the disease (37, 51).

Farmers’ children suffer a high rate of prevalence of infection. This can be explained by those children frequently going to work on their guardians’ farms, with frequent contact with water, which can lead to a high rate of vulnerability to infection when the canals around the farms are infested with the infective stages of the parasite. However, the traders’ children were found to acquire the highest intensity of infection and this is probably due to their extensive exposure to infested water bodies. On the other hand, the pupils whose guardians had a high monthly income were also found to have a high prevalence of infection, while those whose guardians had moderate or low monthly incomes were found to have acquired higher intensities of infection. This result is probably due to the level of exposure to infested water bodies rather than to economic reasons.

## Conclusion

The present study’s findings demonstrate the need for an integrated control program against schistosomiasis including the treatment of all infected children, the implementation of a health education program and measures to combat the intermediate snail host wherever that is possible.
